# Increased Oxidative Stress Induces Apoptosis in Human Cystic Fibrosis Cells

**DOI:** 10.1371/journal.pone.0024880

**Published:** 2011-09-12

**Authors:** Mathilde Rottner, Simon Tual-Chalot, H. Ahmed Mostefai, Ramaroson Andriantsitohaina, Jean-Marie Freyssinet, María Carmen Martínez

**Affiliations:** 1 INSERM, U770, Le Kremlin-Bicêtre, France; 2 Université de Strasbourg, Faculté de Médecine, Strasbourg, France; 3 INSERM, U694, Université d'Angers, Angers, France; University of Birmingham, United Kingdom

## Abstract

Oxidative stress results in deleterious cell function in pathologies associated with inflammation. Here, we investigated the generation of superoxide anion as well as the anti-oxidant defense systems related to the isoforms of superoxide dismutases (SOD) in cystic fibrosis (CF) cells. Pro-apoptotic agents induced apoptosis in CF but not in control cells that was reduced by treatment with SOD mimetic. These effects were associated with increased superoxide anion production, sensitive to the inhibition of IκB-α phosphorylation, in pancreatic but not tracheal CF cells, and reduced upon inhibition of either mitochondrial complex I or NADPH oxidase. CF cells exhibited reduced expression, but not activity, of both Mn-SOD and Cu/Zn-SOD when compared to control cells. Although, expression of EC-SOD was similar in normal and CF cells, its activity was reduced in CF cells. We provide evidence that high levels of oxidative stress are associated with increased apoptosis in CFTR-mutated cells, the sources being different depending on the cell type. These observations underscore a reduced anti-oxidant defense mechanism, at least in part, via diminished EC-SOD activity and regulation of Cu/Zn-SOD and Mn-SOD expressions. These data point to new therapeutic possibilities in targeting anti-oxidant pathways to reduce oxidative stress and apoptosis in CF cells.

## Introduction

Cystic fibrosis (CF) is the most prevalent inherited and lethal disease in the caucasian population. It is due to mutations in the gene encoding the cystic fibrosis transmembrane conductance regulator (CFTR) protein. Expressed in the majority of epithelial cells [1], CFTR, an ATP-dependent membrane glycoprotein, acts as a cAMP-regulated chloride channel [2], and as GSH transporter, the major anti-oxidant of the cell [3], suggesting that CFTR might regulate cellular redox status. More than 1,800 mutations have been identified resulting in a defective CFTR protein [4]. The largely prevalent mutation [*i.e.*, the deletion of residue Phe-508 (ΔF508)] leads to a partial functional trafficking mutant that is capable of conducting chloride, but is prematurely degraded from the endoplasmic reticulum [5].

Absence of functional channel CFTR at the plasma membrane does not permit water flux, leading to dehydrated secretion in all tracts, notably airway and pancreatic tracts [6]–[8], and impaired secretion clearance. Obstruction of tracts results in epithelial destruction and favors the proliferation of bacteria in airways. CF is characterized by chronic inflammation even in absence of pathogens, and by the recruitment of activated neutrophils. The origin of pro-inflammatory mediator production remains obscure and appears to be the consequence of hyperactivation of NF-κB transcription factor and the CFTRΔF508 retention into endoplasmic reticulum (for review see [9]).

Under pathophysiological conditions, activated neutrophils and epithelial cells release highly reactive molecules towards the extracellular space, like reactive oxygen species (ROS) and reactive nitrogen species in order to attack and eliminate invasive pathogens [10], [11]. However, in CF, several evidences show that the defense systems are ineffective. Indeed, mitochondrial levels of ROS are enhanced in CFTR−/− lung epithelial cell line [12] suggesting that, in CF, an increased production of ROS may be associated with cell dysfunction and the incidence of disease. Conversely, in CF airways, levels of nitric oxide (NO) have been described to be low [13] and are associated with a reduction of inducible NO synthase activity [14], [15] that could favor bacterial infection [16].

In addition to excessive oxidative and nitrosative stresses, defective neutralization of ROS can also exacerbate noxious functions in CF. Superoxide anion (O_2_^−^) is dismutated into oxygen and hydrogen peroxide by superoxide dismutase (SOD), an endogenous cellular defense system, which decreases O_2_^−^ levels that damage cells at excessive concentration [17]. Extracellular-SOD (EC-SOD, SOD 3), Mn-SOD (SOD 2) and Cu/Zn-SOD (SOD 1) have been described as potent inhibitors of inflammation [18], [19]. Although no direct evidence has shown the involvement of deregulation of SODs in CF, the fact that EC-SOD is highly expressed in airways and up-regulated in animal models of lung injury [20], it raises the possibility that SODs, and EC-SOD in particular, may play a role in CF. We have previously reported that CF cells displayed an exacerbated apoptosis and NF-κB activation, both contributing to the self-perpetuating inflammatory cycle [21]. In the present study, we investigated the involvement of oxidative stresses in the apoptotic response of CF pancreatic and tracheal cells. For this, we used two pancreatic and tracheal cell lines expressing the wild-type CFTR (PANC-1 and NT-1, respectively) or CFTRΔF508 protein (CFPAC-1 and CFT-2, respectively). Firstly, we investigated whether oxidative stress is implicated in the exacerbated apoptotic response of CF cells using a SOD mimetic and analyzing O_2_^−^ production. Furthermore, we examined the origin of ROS production. Secondly, analysis of SOD expressions and activities were performed.

## Methods

### Reagents

Cell culture reagents, Hank's balanced salt solution (HBSS), and trypsin/EDTA were obtained from Lonza (Verviers, Belgium). Fetal calf serum (FCS) was obtained from Invitrogen (Cergy-Pontoise, France). Actinomycin D (Act D), staurosporine (St), propidium iodide (PI), type I-A RNase A, rotenone, allopurinol, apocynin, and antibody to β-actin were purchased from Sigma-Aldrich (St. Louis, MO). The SOD mimetic, manganese (III) tetrakis (1-methyl-4-pyridyl) porphyrin pentachloride (MnTMPyP) was obtained from Calbiochem (Nottingham, UK). Ripa lysis buffer was provided from Upstate Biotech (Hampshire, UK). Inhibitor of IκB-α phosphorylation (Bay 11-7082) was purchased from BioMol Research Labs, Inc. (Exeter, UK).

### Cell culture and induction of apoptosis

The pancreatic cancer cell line PANC-1 expressing endogenous CFTR and CFPAC-1, presenting the CFTRΔF508 mutation, were purchased from the American Type Culture Collection (Rockville, MD). PANC-1 is a human epithelioid pancreas carcinoma cell line and was grown in DMEM. CFPAC-1 is a human pancreatic adenocarcinoma cell line and was grown in IMDM. The tracheal cell line NT-1, derived from non-CF human fetus, and the CFT-2 cell line, homozygous for the ΔF508 mutation, were a kind gift from Dr. M. Mergey (UMR S893 INSERM, Paris, France) and were grown in DMEM/F12 (1∶1) [21]. All media were supplemented with 10% heat-inactivated FCS, 100 µg/ml streptomycin, and 100 U/ml penicillin. CFPAC-1, NT-1, and CFT-2 cells were incubated in humidified 5% CO_2_ atmosphere at 37°C. PANC-1 cells were cultured at 37°C in a humidified atmosphere of 7.5% CO_2_, as recommended by the American Type Culture Collection. Cell viability was checked by Trypan blue exclusion. Cells were seeded at 7.5×10^4^ cells in T75 flasks. All experiments were carried out when the cells were 80–90% confluent. They were incubated in the presence or absence of Act D (0.5 µg/ml), St (0.33 nM), MnTMPyP (50 µM), Bay 11-7082 (Bay, 7.5 µM), rotenone (5 µM), apocynin (100 µM) or allopurinol (50 µM) for 24 h. All agents were used at concentrations at which no cytotoxicity was observed, as deduced from Trypan blue exclusion.

### Superoxide anion (O2-) determination by electronic paramagnetic resonance (EPR)

After 24 h of apoptosis treatment, cell medium was replaced with deferoxamine-chelated Krebs-Hepes solution containing 1-hydroxy-3-methoxycarbonyl-2,5,5-tetramethylpyrrolidin (CMH; Noxygen, Mainz, Germany) (500 µM), deferoxamine (25 µM), and diethyldithio carbamate (5 µM) under constant temperature (37°C) for 30 minutes. Cells when then scrapped and frozen in plastic tubes and analyzed in a Dewar flask by EPR spectroscopy using a table-top x-band spectrometer Miniscope (MS200; Magnettech, Berlin, Germany), as previously described [22]. Values are expressed as amplitude of signal per protein concentration.

### Determination of hypodiploid DNA

After treatments, culture medium was removed from cells growing in monolayers; adherent cells were trypsinized, detached, combined with floating cells from the original culture medium, and centrifuged. Cells were then fixed in 70% ethanol for at least 4 h at 4°C and washed once in 1 mM HBSS Ca^2+^ before resuspension for 10 min in a solution containing type I-A RNase A (0.05 mg/ml) in HBSS containing 1 mM Ca^2+^ at 37°C. PI was then added at a final concentration of 0.1 mg/ml, as previously described [21]. After 15 min in the dark at room temperature, samples were analyzed by flow cytometry using a FACScan flow cytometer (Becton Dickinson, San Jose, CA). Data acquisition (10,000 events in each case) and analysis were conducted using CELLQuest software (Becton Dickinson). The forward light scatter setting was E-01.

### Western blot analysis

After incubation with apoptosis-inducing agents for 24 h, cells were scrapped in the presence of 400 µl of Ripa buffer with 10 µg/ml leupeptin, 10 µg/ml pepstatin, 10 µg/ml aprotinin, and 1 mM phenylmethanesulfonylfluoride. Samples containing 20 µg proteins (Bio-Rad protein assay kit) were separated on 10% SDS-PAGE. Separated proteins were then blotted onto Hybond-ECL nitrocellulose membrane (Amersham Biosciences, Buckinghamshire, UK). Blots were probed with antibodies against Mn-SOD, Cu/Zn-SOD and EC-SOD (Stressgen, MI), and developed with horseradish peroxidase-conjugated secondary antibody. Bound antibodies were revealed by chemiluminescence (Pierce, Rockford, IL); ß-actin staining was used as control. Enzyme levels were determined by densitometry analysis and were normalized with respect to ß-actin.

### Determination of SOD activities

Analysis of SOD activities were performed according to the manufacturer's instructions (Stressgen, MI). Briefly, cells were cultured in the absence or in the presence of Act D (0.5 µg/ml) or St (0.33 nM), and after 24 h, cells were washed, detached with trypsin and washed with ice cold phosphate buffer saline. EC-SOD activity measurement was performed using culture supernatant and Cu/Zn-SOD and Mn-SOD using cell lysate. Also, Cu/Zn-SOD was isolated by adding ice-cold chloroform/ethanol (37.5/62.5 (v/v)) and its activity measured. Absorbance was read at 405 nm for 10 min at room temperature. Data were expressed as µg of protein/µl.

### Statistical analysis

Data are represented as mean ± SEM; *n* represents the number of experiments. Statistical analysis was carried out using Student's *t* test or non-parametric Mann-Whitney *U* test. Differences were considered statistically significant at a value of *p*<0.05.

## Results

### The SOD mimetic reduces the increased sensitivity to apoptogenic agents in cells with CFTR dysfunction

**Figure 1:**
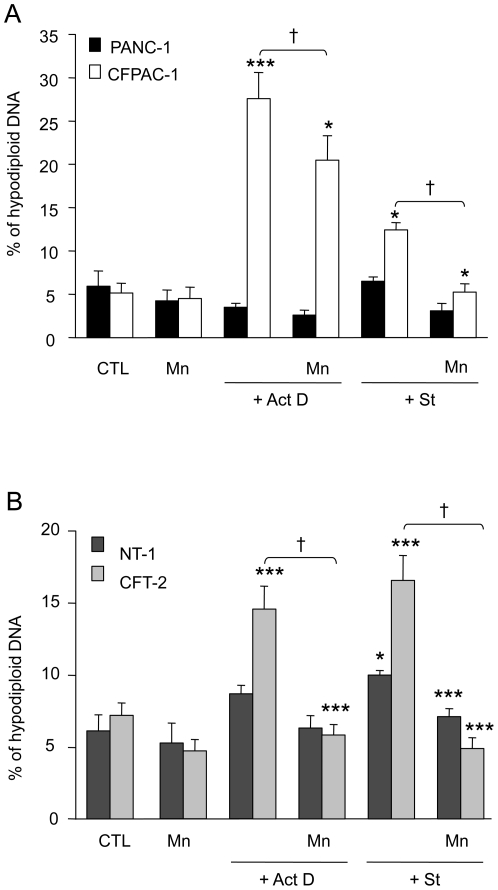
The SOD mimetic MnTMPyP reduces the increased sensitivity to apoptogenic agents of cells with CFTR dysfunction. (A) At confluence, PANC-1 (n = 6, black bars) and CFPAC-1 (n = 6, open bars) and (B) NT-1 (n = 6, dark gray bars) and CFT-2 (n = 6, light gray bars) cells were treated with MnTMPyP (Mn) for 30 min before treatment with actinomycin D (Act D) or staurosporine (St) for 24 h, or without any treatment (CTL). Cells were permeabilized with 70% ethanol and hypodiploid DNA was quantified by the use of propidium iodide. * *p*<0.05, *** *p*<0.001 significantly different from respective control cells; † *p*<0.05 significantly different between in the absence and in the presence of Mn.

Pancreatic and tracheal cells were incubated in the absence and in the presence of the SOD mimetic MnTMPyP (50 µM), 30 min before treatment with pro-apoptotic agents for 24 h. Staining with PI revealed nuclei with hypodiploid DNA (sub-G1 peak) corresponding to apoptotic cells, measured by flow cytometry. As previously described [21], CF cells displayed exacerbated apoptosis in the presence of Act D or St (Fig. 1, A and B). Higher concentrations of apoptotic agents did not induce further increase of apoptosis, but an enhanced necrosis was observed (near of 60%). MnTMPyP treatment had no effect on basal apoptosis which was not significantly different between normal cells or cells with CFTR dysfunction (Fig. 1, A and B). Interestingly, MnTMPyP was able to decrease Act D- and St-induced apoptosis in CF cells. Indeed, MnTMPyP decreased hypodiploid DNA content by 29% and 58% in Act D- and St-treated CF pancreatic cells, respectively (Fig. 1A). Similar results were obtained in tracheal cells, MnTMPyP inhibited apoptosis by 62% and 73% in Act D- and St-treated CFT-2 cells, respectively (Fig. 1B). These results suggest that oxidative stress is involved in the induction of apoptosis in CF cells.

### O_2_^−^ production in normal and CF cells

**Figure 2:**
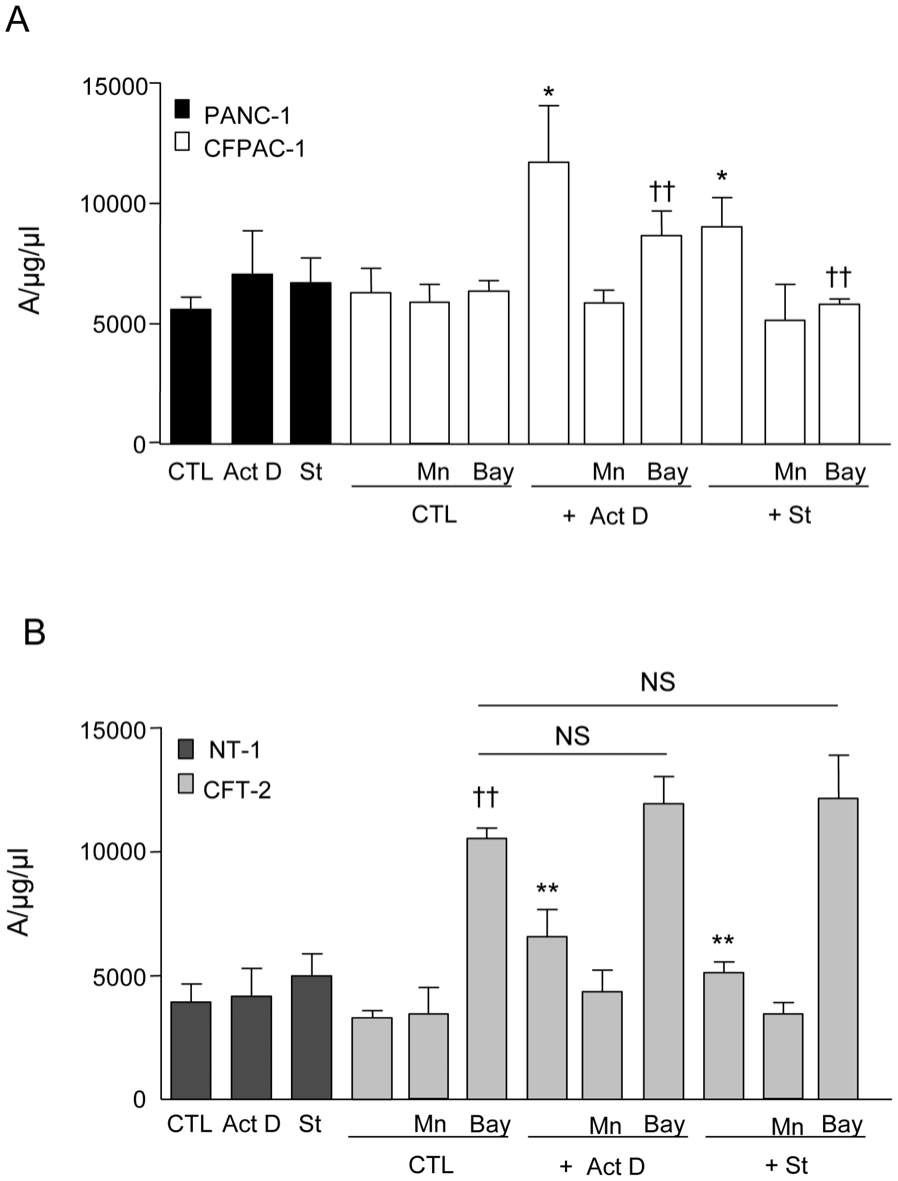
Superoxide anion production by pancreatic and tracheal cell lines after pro-apoptotic treatments. (A) At confluence, PANC-1 (n = 5, black bars) and CFPAC-1 (n = 5, open bars), and (B) NT-1 (n = 5, dark gray bars) and CFT-2 (n = 5, light gray bars) cells were treated with the SOD mimetic MnTMPyP (Mn) for 30 min or with the inhibitor of phosphorylation of IκB-α, Bay-11702 (Bay) for 30 min before treatment with actinomycin D (ActD) or staurosporine (St) for 24 h, or without any treatment (CTL). Then, cells were incubated in the presence of superoxide anion spin trap and quantification of the amplitude of the superoxide anion-CMH complex signal was performed by electronic paramagnetic resonance. Values are expressed as units per protein concentration (µg/ml). * *p*<0.05, ** *p*<0.01 significantly different from respective control cells; †† *p*<0.01 significantly different from in the absence of Bay. NS = not significant.

All types of cells exhibited an EPR feature of signals derived from CMH-O_2_^−^ complex. Measurement of O_2_^−^ production shows that, in pancreatic and tracheal normal cells, apoptotic treatment did not induce significant changes in O_2_^−^ levels (Fig. 2, A and B). By contrast, Act D- or St-treated CF cells displayed an increase of O_2_^−^ levels (Fig. 2, A and B). Treatment with the SOD mimetic MnTMPyP abolished the increase in O_2_^−^ levels evoked by Act D or St in CF cells (Fig. 2, A and B). To determine the sources of O_2_^−^ production involved in the induction of apoptosis in CF cells, both pancreatic and tracheal CF cells were incubated in the presence of inhibitors of xanthine oxidase (allopurinol), NADPH oxidase (apocynin) or mitochondrial complex I (rotenone), and Act D-induced apoptosis was evaluated. Apoptosis induction was independent of xanthine oxidase in both pancreatic and tracheal cells. In contrast, rotenone reduced apoptosis in pancreatic CF cells and apocynin in tracheal CF cells (Table 1).

**Table 1:** Effects of inhibition of NADPH oxidase by apocynin, xanthine oxidase by allopurinol and mitochondrial complex I by rotenone on actinomycin D-induced apoptosis in pancreatic (CFPAC-1) and tracheal (CFT-2) cystic fibrosis cells (n = 5).

	CFPAC-1	CFT-2
**Actinomycin D**		
**Apocynin**	0.4±0.6%	14±0.02%*
**Allopurinol**	4.1±0.4%	11±0.1%
**Rotenone**	12±0.5%*	1.1±0.4%

Data are expressed in percentage of inhibition of actinomycin D-induced apoptosis.

*p<0.05.

Because, in CF cells, NF-κB pathway is activated under basal as well as apoptotic conditions [21], we have investigated the effects of inhibition of IκB-α phosphorylation on O_2_^−^ production, using Bay 11-7082. Interestingly, when Iκ-Bα phosphorylation was inhibited in pancreatic CF cells, basal O_2_^−^ production was not modified but the increase in O_2_^−^ generation induced by apoptotic agents was reduced (Fig. 2A). Surprisingly, in tracheal CF cells, inhibition of Iκ-Bα phosphorylation induced a strong increase in O_2_^−^ production and blunted the response evoked by apoptotic treatment (Fig. 2B).

### Expression of SOD in normal and CF cells

**Figure 3:**
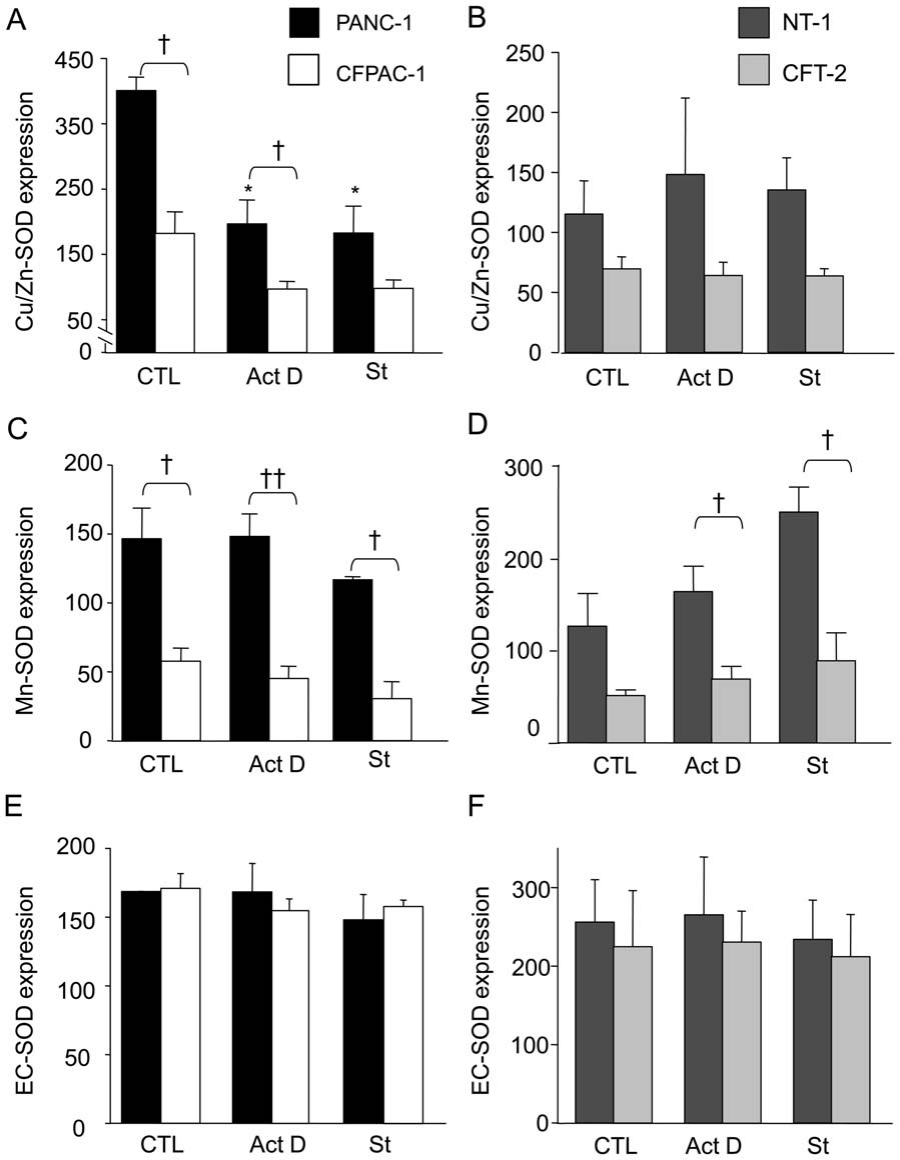
Expression of SOD in pancreatic and tracheal cells. At confluence, PANC-1 (n = 5, black bars) and CFPAC-1 (n = 5, open bars), NT-1 (n = 5, dark gray bars) and CFT-2 (n = 5, light gray bars) cells were treated with actinomycin D (Act D) or staurosporine (St) for 24 h, or without any treatment (CTL). Five determinations yielding similar results were performed. A β-actin control was included. Western-Blot were performed for Cu/Zn-SOD (A, B), for Mn-SOD (C, D) and for EC-SOD (E, F). SOD expressions were quantified by densitometric analysis and measurements were normalized with respect to β-actin. Densitometry values are given as mean ± SEM * *p*<0.05 significantly different from respective control cells; † *p*<0.05, †† *p*<0.01 significantly different between both types of cells.

As shown in Fig. 3A–D, expression of both Cu/Zn-SOD and Mn-SOD was down-regulated in CF cells. On the one hand, pro-apoptotic stimuli significantly decreased Cu/Zn-SOD expression in normal pancreatic cells (PANC-1) but not in normal tracheal cells (NT-1). On the other hand, pro-apoptotic stimuli increased Mn-SOD expression in NT-1 cells but not in PANC-1. Of note was that pro-apoptotic stimuli had no effect on either Cu/Zn-SOD or Mn-SOD in CF cells. Concerning EC-SOD, no difference in expression was observed in both normal and CF cells (Fig. 3, E and F). In addition, pro-apoptotic treatment had no effect on EC-SOD expression.

### SOD activity in normal and CF cells

**Figure 4:**
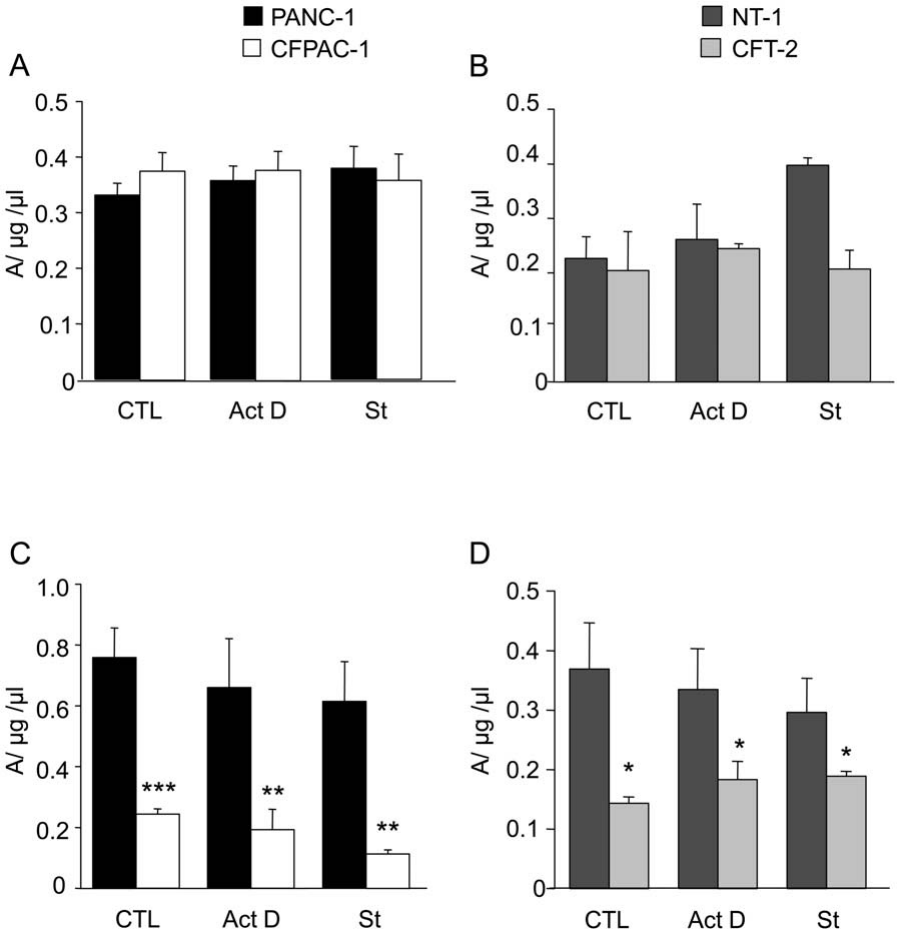
Activity of SODs in pancreatic and tracheal cells. At confluence, PANC-1 (n = 5, black bars) and CFPAC-1 (n = 5, open bars), NT-1 (n = 5, dark gray bars) and CFT-2 (n = 5, light gray bars) cells were treated with actinomycin D (Act D) or staurosporine (St) for 24 h, or without any treatment (CTL). Activity of both Cu/Zn-SOD and Mn-SOD are measured in A and B, and activity of EC-SOD in C and D. Enzymatic activity is expressed in absorbance units (A) per total protein concentration (µg/µl). † *p*<0.05, †† *p*<0.01, ††† *p*<0.001 significantly different between both types of cells.

Activities of intracellular SODs (Cu/Zn-SOD and Mn-SOD) and EC-SOD were measured in pancreatic and tracheal cells (Fig. 4). No differences were observed in Cu/Zn-SOD and Mn-SOD activities between normal and CF cells. Interestingly, activity of EC-SOD was lower in CF cells than in normal cells. Pro-apoptotic treatments did not modify SOD activities.

## Discussion

It has been suggested that excessive ROS production accounts for a variety of the degenerative processes of some human diseases due to their deleterious effect to target cells [11], [23], [24]. The data reported here show that increased susceptibility of apoptosis of pancreatic and tracheal CF cells is associated with enhanced ROS production, because SOD mimetic reduced apoptosis. Also, inhibition of NF-κB pathway strongly reduced O_2_^−^ production in CF pancreatic cells suggesting a key role of this pathway in the regulation of oxidative stress. Furthermore, expression of anti-oxidant defense enzymes, Cu/Zn-SOD and Mn-SOD, was down-regulated in CF cells whereas their activities were not affected by CFTR mutation or by apoptotic treatment. In addition, EC-SOD activity, but not its expression, was reduced in CF cells when compared to normal cells. Altogether, these results suggest that disruption of the balance between ROS generation via NF-κB inhibitor-sensitive pathway and anti-oxidant defense may account for the sustained apoptosis and pro-inflammatory profile observed in CF cells.

We have previously shown that both Act D and St induced apoptosis but not necrosis in CF cells as demonstrated by double staining Annexin V/propidium iodide and TUNEL assays [21]. In addition, no apoptosis was observed in control cells under the same experimental conditions. This was not due to a delayed apoptotic response, since hypodiploid DNA was measured in all types of cells after 12 h, 24 h, and 48 h of apoptosis stimulation and DNA fragmentation was higher in CF cells than in control cells [21]. In addition, new experiments performed in control cells using a higher concentration of apoptotic agents showed no increase of apoptosis, but an enhanced necrosis (near of 60%).

ROS are important mediators of apoptosis mainly in vascular and epithelial cells, which subsequently initiate a series of local chemical reactions and genetic alterations resulting in an amplification of the initial ROS-mediated tissue damage and/or cytotoxicity [25]. It is estimated that normal levels of ROS are efficiently detoxified by endogenous enzymatic ROS scavengers such as SOD [26]. However, under conditions associated with excessive ROS production, the rate of ROS generated can exceed the capacity of anti-oxidant defense mechanisms to scavenge ROS and prevent deleterious ROS-evoked reactions. Concerning CF, it has been shown that endogenous ROS and lipid peroxidation levels are higher in CFTR−/− lung when compared to wild-type (CFTR+/+) in basal conditions, despite a strong enzymatic antioxidant expression involving SOD, indicating a constitutive redox imbalance [27]. Also, increased oxidative stress is responsible to defective autophagy in CF cells resulting in the accumulation of misfolded mutant CFTR protein [28]. Here, we provide evidence that O_2_^−^ mediated exacerbated apoptosis in CF cells since SOD mimetic, MnTMPyP, was able to reduce apoptosis induced by Act D and St. It has been reported that, in hepatocytes, oxidative stress induced by O_2_^−^ activates caspases and evokes mitochondria-mediated apoptosis through the involvement of the Bcl-2 family proteins [29]. Regarding the source of O_2_^−^, whereas mitochondria seem to play a role in pancreatic CF cells, inhibition of NADPH oxidase with apocynin reduced apoptosis in tracheal CF cells indicating the implication of NADPH oxidase. These results are in accordance with those showing that the main source of O_2_^−^ in pancreatic and tracheal cells is mitochondria and NADPH oxidase, respectively. Thus, in pancreatic cells, mitochondrial complex I and III are involved in ROS generation leading to apoptosis induction [30]. In tracheal epithelial and smooth muscle cells, the increase of O_2_^−^ production via the activation of NADPH oxidase may exacerbate pulmonary inflammation [31], [32]. However, we cannot exclude that other sources of O_2_^−^ might be implicated in the mechanisms leading to apoptosis in CF cells. Indeed, inhibition of complex I with rotenone reduced Act D-induced apoptosis of pancreatic CF cells about ∼12% whereas the SOD mimetic reduced it ∼30%. Similar results were observed in tracheal CF cells. These results suggest that probably other sources of O_2_^−^ are involved in the induction of apoptosis in CF cells.

Interestingly, (i) basal O_2_^−^ production was similar in all cell types, (ii) apoptotic treatment did not modify O_2_^−^ production in normal cells, (iii) in CF cells, apoptotic treatment enhanced O_2_^−^ production, (iv) inhibition of NF-κB pathway reduced apoptosis-induced O_2_^−^ production in pancreatic CF cells, and (v) in tracheal CF cells, the NF-κB pathway seems to regulate basal production of O_2_^−^. We have previously shown that NF-κB pathway controls apoptosis and inflammation in CF cells [21]. Altogether these results indicate that NF-κB pathway, in part via stimulation of oxidative stress, plays an important role in mediating both apoptosis and inflammation in CF cells. Unexpectedly, inhibition of NF-κB pathway, in the absence of apoptosis inducers, elicited a strong increase on O_2_^−^ production only in tracheal CF cells, suggesting a beneficial role for NF-κB activation in tracheal, but not pancreatic, CF cells. It is possible that differences in the regulation of basal O_2_^−^ production by NF-κB are related to the different profile of pro-inflammatroy secretome of both types of cells [21] or the regulation of NADPH oxidase activity by the NF-κB pathway [32], [33]. Under these conditions, apoptotic stimuli failed to further enhance O_2_^−^ production, probably because the system was already exhausted upon blockade of NF-κB pathway. This also strengthens the hypothesis that an exacerbated negative control of O_2_^−^ production via NF-κB pathway under normal conditions. Indeed, dual effects of NF-κB by exerting either protective or deleterious effect have been reported depending on the conditions [34].

EC-SOD is highly expressed in airways and up-regulated in animal models of lung injury [20]. These results raise the possibility that SODs may play a role in CF. This hypothesis was further assessed by looking at both expressions and activities of three isoforms of SODs. Expression of Cu/Zn-SOD and Mn-SOD isoforms was reduced in CF cells when compared to normal cells even though their activities were not modified. The fact that apoptotic treatments exerted differential effects on the two isoforms in the normal but not in CF cells suggests that these stimuli might not play a major role in controlling the expression of these enzymes in CF. By contrast, EC-SOD expression was not modified but its activity was reduced in CF cells. Thus, although an insufficient expression of SODs could account for an increased level of O_2_^−^, it is most likely that the reduction in EC-SOD activity plays an essential role in the elevation on O_2_^−^ levels. In agreement with the present results, Madarasi et al. [35] have shown that in plasma from patients with CF, SOD activities were significantly lower when compared with healthy subjects. Also, reduced activity of Cu/Zn-SOD has been described in mononuclear, polymorphonuclear and red cells in CF patients [36], [37]. Although EC-SOD has not been studied in CF, it has been shown that EC-SOD overexpression attenuates endotoxin-induced acute lung injury [38] and EC-SOD knock-out mice are more sensitive to pulmonary inflammation than wild type mice [39] suggesting that EC-SOD limits injury in response to many pulmonary insults. Altogether, these results suggest a reduced anti-oxidant defense mechanism in CF cells, at least in part, via diminished EC-SOD activity.

**Figure 5:**
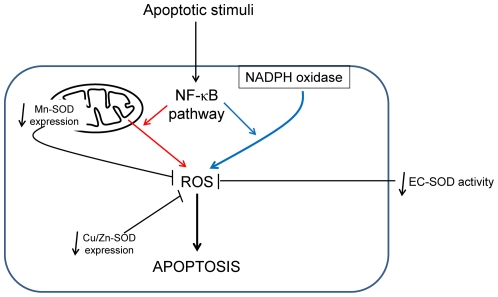
Representative schema showing that, in CF cells, increased apoptosis and NF-κB activation are associated with high levels of oxidative stress. Apoptotic stimuli seem activate NF-κB pathway which regulate reactive oxygen species (ROS) production. Whereas in pancreatic CF cells ROS are derived from mitochondria (red lines), in tracheal CF cells ROS are produced mainly by NADPH oxidase (blue lines). In addition, in both types of CF cells, a reduced anti-oxidant defense mechanism at least in part via diminished EC-SOD activity and reduced Cu/Zn-SOD and Mn-SOD expressions lead to exacerbate oxidative stress.

In conclusion, we provide evidence that, in CFTR mutated cells, the link between increased apoptosis and NF-κB activation associated with inflammation results in high levels of oxidative stress (Fig. 5). These observations further underscore a reduced anti-oxidant defense mechanism at least in part via diminished EC-SOD activity and subtle regulation of Cu/Zn-SOD and Mn-SOD expression and activities. These data point to new therapeutic possibilities targeting anti-oxidant pathways to reduce oxidative stress and apoptosis in CF cells, and thus to limit pro-inflammatory response in this pathology.
